# Comparative analysis of cecal microbiota and metabolites in relation to growth performance of Tibetan and Hu sheep

**DOI:** 10.3389/fmicb.2025.1725706

**Published:** 2026-01-26

**Authors:** Feifei Yang, Lei Yang, Jinping Shi, Zhixiong Tang, Quanlu Meng, Zhengwu Pi, Haoke Li, Ting Liu, Changji Zhang, Shuru Cheng

**Affiliations:** 1 College of Animal Science and Technology, Gansu Agricultural University, Lanzhou, China; 2 Dingxi Vocational and Technical College, Dingxi, China; 3 School of Animal Science and Technology, Ningxia University, Yinchuan, China; 4 College of Biological and Architectural Engineering, Baoji Vocational and Technical College, Baoji, China

**Keywords:** cecum, intestinal microbiota, metabolomics, production performance, sheep

## Abstract

The gastrointestinal microbiota play a crucial role in the growth and development of sheep. However, most existing studies have focused on the rumen microbiota, while comparatively little attention has been given to the cecum—the primary site of hindgut fermentation—and its metabolic functions. To investigate the potential influence of cecal microbiota and their metabolites on growth performance, we selected healthy male lambs of Tibetan and Hu sheep breeds at 3 months of age with similar body weights (19.55 ± 1.51 kg). After 3 months of feeding under identical conditions, the lambs were slaughtered, and cecal contents were analyzed using 16S rRNA gene sequencing and liquid chromatography–mass spectrometry (LC–MS). Results showed that Hu sheep exhibited significantly superior growth performance [body weight, average daily gain (ADG), and body size] compared to Tibetan sheep, accompanied by higher cecal concentrations of acetic, propionic, and butyric acids (*p* < 0.05). Microbial diversity analysis revealed that Firmicutes and Bacteroidetes were the dominant microbial phyla in the cecum of both breeds. At the genus level, the relative abundances of *norank_f__Lachnospiraceae*, *UCG-005*, and *norank_f__Eubacterium_coprostanoligenes_group* were higher in Tibetan sheep, whereas *Rikenellaceae_RC9_gut_group* and *Bacteroides* predominated in Hu sheep. Metabolomic profiling identified 986 differentially abundant metabolites, primarily enriched in nucleotide, tryptophan, and arachidonic acid metabolism pathways. Notably, five bacterial genera (*norank_f__Christensenellaceae, Negativibacillus, Christensenellaceae_R-7_group, norank_o__Clostridia_UCG-014*, and *Prevotellaceae_NK3B31_group*) and five key metabolites (4-O-(indole-3-acetyl)-D-glucopyranose, 5Alpha-cyprinol, 2-Hydroxyphenylacetic acid, Myrianthic acid, and Indole-3-carboxylic acid-O-sulfate) were identified as closely associated with growth traits. Correlation analyses among microorganisms, metabolites, and growth performance revealed significant positive associations between these bacterial genera, metabolites, and growth traits. Collectively, these findings suggest that specific gut microbes and metabolites synergistically enhance host growth and development by modulating energy metabolism pathways. This study provides novel insights into the cooperative mechanisms through which gut microbiota and metabolites regulate growth performance in sheep.

## 1 Introduction

China accounts for approximately 25% of the world’s sheep breeds, constituting one of the richest repositories of ovine genetic diversity (Cheng et al., 2020). Among China’s indigenous breeds, Tibetan and Hu sheep attract considerable attention owing to their distinct advantageous traits. With long-term adaptation to high-altitude, hypoxic, and cold environments, Tibetan sheep exhibit strong stress tolerance and distinctive meat quality (Liu et al., 2022; Zhang et al., 2022). Hu sheep, noted for rapid growth and high fecundity, are a mainstay in modern intensive production systems (Chen et al., 2022). In recent years, crossbreeding between Tibetan and Hu sheep has been increasingly implemented to combine their respective advantages in adaptability and productivity (Wei L. et al., 2025). However, the gut microbial mechanisms underlying these traits remain poorly understood. Recent advances in microbiome research indicate that gut microbiota play central roles in nutrient metabolism, immune regulation, and growth (Li K. et al., 2022). Therefore, elucidating breed-specific differences in gut microbiota composition and function between Tibetan and Hu sheep is essential. Furthermore, determining how microbial modulation affects growth performance is an emerging priority for sheep breeding and health-oriented production.

The gastrointestinal microbiota and their metabolites are central regulators of host growth and development (Lahiri et al., 2019). In ruminants, rumen microbes receive considerable attention due to their direct effects on production performance. For instance, Ghazanfar et al. (2015) reported that enhancing rumen microbial activity improves growth. Xue et al. (2018) proposed that characterizing the rumen core and pan-microbiome may provide avenues to increase milk yield. However, most research has focused on the rumen, whereas the cecum—another major site of fermentation—has received comparatively little attention. The gut is central to health, supporting digestion, absorption, metabolism, and immunity (Li et al., 2019). As a principal site of hindgut fermentation, the cecum contributes to nutrient metabolism and immune homeostasis and influences disease processes through microbiota–host interactions (Zhen et al., 2025). Volatile fatty acids (VFAs) generated from dietary fiber fermentation fuel intestinal epithelial cells and regulate systemic energy metabolism (Ma et al., 2021). They also modulate immune cell activity, T cell differentiation, and inflammatory responses via VFA-mediated pathways and TLR2–MyD88–NF-κB signaling, thereby shaping barrier function and systemic immune homeostasis (Kesika et al., 2021; Yan et al., 2024). Gut microbiota dysbiosis is associated with impaired barrier function, systemic inflammation, and increased risk of extraintestinal disease. Conversely, dietary interventions and probiotic supplementation that modulate the microbiota may improve host health (Liu et al., 2024). In ruminants, the cecum—a vital component of the hindgut—plays a central role in fiber fermentation, mucosal immune regulation, and metabolism. Cecal dysbiosis–associated malnutrition has been linked to adverse outcomes, including abortion in pregnant animals and reduced immune competence (Godoy-Vitorino et al., 2012). Studies indicate that the cecal microbiota in Yunshan Black goats are positively correlated with immune markers, including TLR-3, TLR-4, and IFN-γ, supporting a role in mucosal immune regulation (Fu et al., 2025). Further evidence suggests that cecal microbial dysbiosis may trigger inflammatory responses and compromise intestinal barrier function (Wu et al., 2023). In cattle, appropriate probiotic supplementation increases cecal microbial richness and improves growth performance (Fuerniss et al., 2022). Under cold stress, Hulunbuir sheep exhibit cecal microbiota shifts characterized by increased propionate and butyrate metabolism (Cheng et al., 2025).

This study investigated Tibetan and Hu sheep, beginning with an assessment of growth performance. Subsequently, cecal contents were collected for VFA quantification, 16S rRNA gene sequencing, and metabolomic profiling. Integrated microbiome–metabolome analyses identified key microbial taxa and functional metabolites linked to growth performance. These findings provide a theoretical framework for leveraging microbial modulation to enhance production performance in sheep.

## 2 Materials and methods

### 2.1 Animal ethics statement

All experimental protocols were approved by the Animal Ethics Committee of Gansu Agricultural University (Lanzhou, China; Approval Number: GSAU-Eth-AEW-2024-032) and were carried out in strict accordance with applicable ethical guidelines and regulations.

### 2.2 Experimental design and sample collection

The experiment was conducted in Guanghe County, Gansu Province, China. A total of 100 healthy male lambs, including Tibetan sheep (*n* = 50) and Hu sheep (*n* = 50), with an initial body weight of 19.55 ± 1.51 kg at 3 months of age, were selected. Throughout the experimental period, all lambs were housed individually under standardized management conditions, fed a uniform diet, and provided ad libitum access to feed and water (Supplementary Table S1). At 6 months of age, 12 lambs from each breed were randomly selected (a total of 24 animals). After a 24 h fasting period and 12 h water withdrawal, these lambs were humanely slaughtered in accordance with the institutional animal welfare guidelines. Immediately after slaughter, the cecal contents were collected into sterile 5 mL cryovials, rapidly frozen in liquid nitrogen, and stored at −80 °C until analysis. These samples were used for 16S rRNA gene sequencing, untargeted metabolomic analysis, and the determination of VFA concentrations.

### 2.3 Determination of volatile fatty acids in cecal contents

VFA concentrations were measured with a gas chromatograph (Agilent 7890B; Palo Alto, CA, USA) following the method of Shi et al. (2023). Cecal contents were centrifuged at 5,400 rpm for 10 min at 4 °C. Then, 1 mL of supernatant was transferred to a 1.5 mL tube. The same procedure was applied to the appendix contents. Each sample was mixed with 0.2 mL of 25% metaphosphoric acid containing the internal standard 2-ethylbutyric acid (2-EB), vortexed thoroughly, and incubated on ice for 30 min. The mixture was centrifuged at 10,000 rpm for 10 min at 4 °C. The supernatant was filtered through a 0.22 μm organic-phase membrane and stored in a 2-mL amber vial for gas-chromatography analysis. The injector and detector were set to 250 °C and 260 °C, respectively. The oven temperature program was 60 °C for 1 min, ramped at 5 °C/min to 115 °C, then at 15 °C/min to 180 °C.

### 2.4 16S rRNA gene sequencing and bioinformatics analysis

Total microbial genomic DNA was extracted using the E.Z.N.A.® Soil DNA Kit (Omega Bio-tek, Norcross, GA, USA) following the manufacturer’s instructions. DNA integrity was assessed by agarose gel electrophoresis (1%), and concentration and purity were measured with a NanoDrop 2000 spectrophotometer (Thermo Scientific, USA). Using extracted DNA as template, the 16S rRNA V3–V4 region was amplified with primers 338F (5′-ACTCCTACGGGAGGCAGCAG-3′) and 806R (5′-GGACTACHVGGGTWTCTAAT-3′) (Liu et al., 2016), each carrying a sample-specific barcode. Purified amplicons were prepared into libraries with the NEXTFLEX® Rapid DNA-Seq Kit and sequenced as paired-end reads on an Illumina NextSeq 2000 platform. Raw paired-end reads were quality-filtered with fastp (v0.19.6) (Chen et al., 2018), merged with FLASH (v1.2.11) (Magoč and Salzberg, 2011), and denoised in QIIME 2 (Bolyen et al., 2019) to generate amplicon sequence variants (ASVs). Taxonomic assignment was performed against the SILVA 16S rRNA database (release 138) (Quast et al., 2013) using the Naive Bayes classifier in QIIME 2. Functional inference from 16S profiles was performed with PICRUSt2 (v2.2.0) (Douglas et al., 2020) against the Kyoto Encyclopedia of Genes and Genomes (KEGG) pathways.

### 2.5 Non-targeted metabolomics sequencing and bioinformatics analysis

Weigh 50 mg of cecal contents into a 2 mL centrifuge tube. After thawing the samples at 4 °C, add one 6-mm grinding bead and 400 μL of extraction solvent containing the internal standard L-2-chlorophenylalanine (0.02 mg/mL). Samples were cryo-ground at −10 °C and 50 Hz for 6 min, then ultrasonicated at 5 °C and 40 kHz for 30 min. Extracts were held at −20 °C for 30 min and centrifuged at 13,000 *g* for 15 min at 4 °C. The supernatant was filtered and transferred to an autosampler vial with a fixed insert for analysis. Equal aliquots from all samples were pooled to generate quality-control (QC) samples for evaluating analytical reliability and repeatability. Metabolite profiling was performed by LC–MS on a UHPLC–Q Exactive Orbitrap system (Thermo Fisher Scientific, MA, USA). Raw LC–MS files were processed in Progenesis QI (Waters, Milford, USA) for peak detection and alignment; metabolite annotation used HMDB (v4.0), METLIN (v5), and public databases. Differential metabolites were prioritized using variable importance in projection (VIP) from OPLS-DA and Student’s t-test *p* values; features with VIP > 1 and *p* < 0.05 were considered differential. Associated pathways were annotated against KEGG, and enrichment was tested with SciPy (scipy.stats). Pathways most relevant to the experimental treatments were evaluated using Fisher’s exact test.

### 2.6 Data statistics and analysis

Statistical analyses were conducted with IBM SPSS Statistics 27.0 (IBM Corp., Armonk, NY, USA). Data are presented as mean ± standard error of the mean (SEM) and conducted a significance analysis using an independent samples t-test. O2PLS analyses, heat maps, STAMP plots, and bar charts were generated with OmicShare Tools,^1^ OmicStudio cloud tools,^2^ and the Chiplot platform.^3^ Metabolite Pathway Exploration (MPEA) was performed with MetOrigin.^4^ Statistical graphics were created in GraphPad Prism 9.5.1 (GraphPad Software, USA).

## 3 Results

### 3.1 Comparison of growth performance

As shown in Table 1, Hu sheep exhibited superior growth performance compared to Tibetan sheep. Hu sheep significantly outperformed Tibetan sheep in body weight, average daily weight gain (ADG), height, body length, and chest circumference (*p* < 0.001). While Hu sheep had a slightly larger cannon bone circumference, this difference was not statistically significant (*p* > 0.05).

**Table 1:** Comparative analysis of production performance.

Item	Hu	Tibetan	*p*-Value
Weight, kg	46.93 ± 0.520	38.07 ± 0.240	<0.001
ADG, kg/d	0.30 ± 0.005	0.22 ± 0.006	<0.001
Height, cm	73.14 ± 0.840	54.50 ± 1.980	<0.001
Body oblique length, cm	76.69 ± 0.890	60.24 ± 0.690	<0.001
Chest circumference, cm	100.19 ± 1.520	68.90 ± 0.770	<0.001
Pipe circumference, cm	7.65 ± 0.300	7.19 ± 0.310	0.303

Data are presented as mean ± SEM.

### 3.2 Volatile fatty acids analysis in the cecum

**Figure 1:**
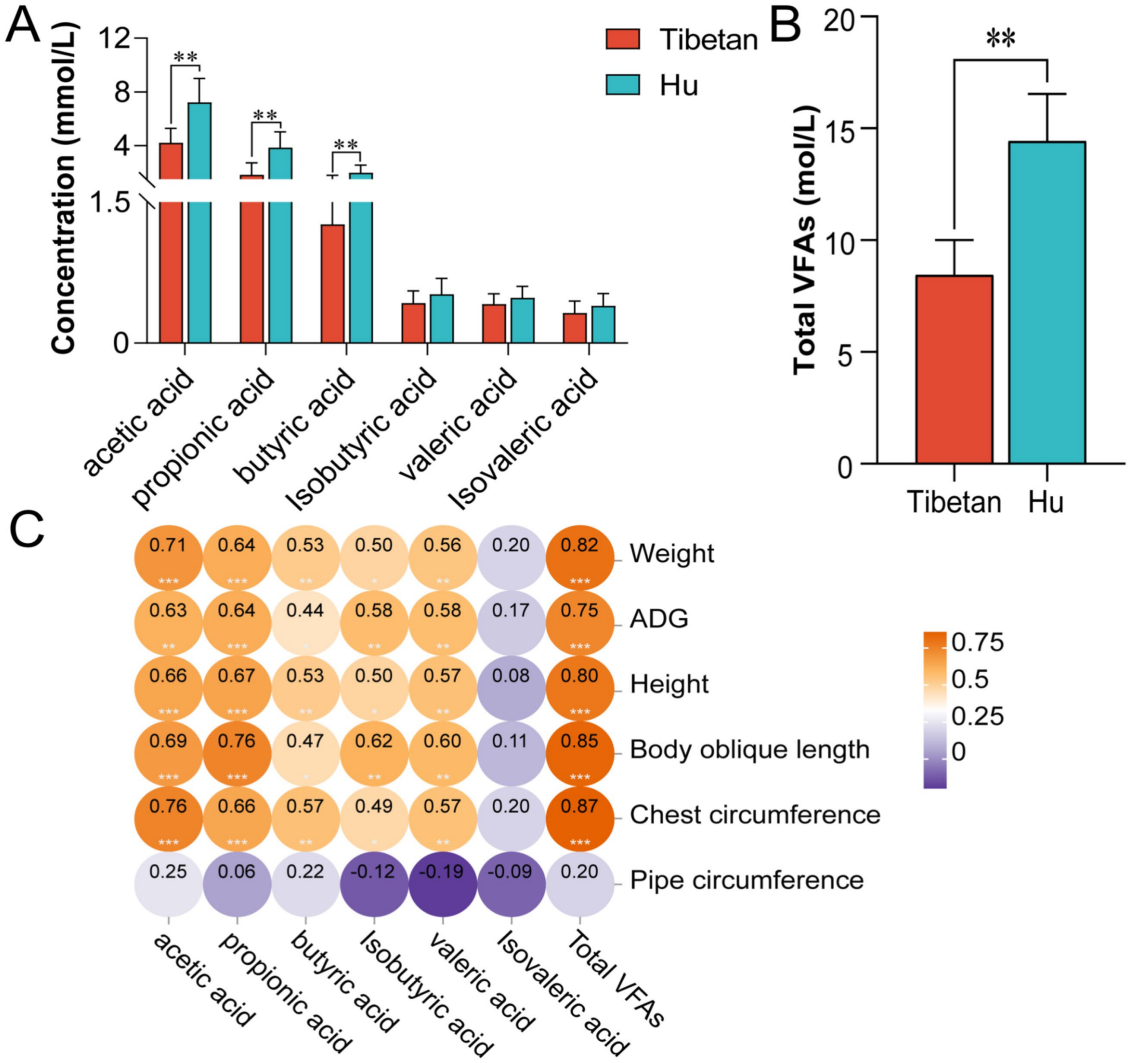
Comparative analysis of cecal volatile fatty acid profiles between Tibetan and Hu sheep. **(A)** Concentrations of hexanoic acid, propionate, butyrate, isobutyrate, valerate, and isovalerate in cecal contents. **(B)** Total VFAs content in cecal contents. **(C)** Correlation between VFAs and sheep growth traits. Data are presented as mean ± SEM. *p < 0.05, **p < 0.01, and ***p < 0.001.

The experiment evaluated changes in the concentrations of six major VFAs in the cecum. Results showed that Hu sheep exhibited significantly higher concentrations of acetic acid, propionic acid, and butyric acid compared to Tibetan sheep (*p* < 0.05), while concentrations of isobutyric acid, valeric acid, and isovaleric acid were slightly higher than those in Tibetan sheep, though the differences were not significant (*p* > 0.05; Figure 1A). Additionally, the total VFAs concentration in the cecum of Hu sheep was significantly higher than that in Tibetan sheep (Figure 1B). Correlation analysis revealed that total VFAs were significantly positively correlated with body weight, daily weight gain, and body height in sheep (*p* < 0.05; Figure 1C).

### 3.3 Microbial diversity and sequencing overview of cecal microbiota

A total of 24 cecal content samples were subjected to 16S rRNA gene sequencing, yielding 1,741,884 raw reads. After quality control, chimera removal, denoising, and filtering of low-quality sequences, 1,700,604 high-quality reads were retained. The average number of high-quality sequences per sample was 70,858, with an average alignment rate of 97.63%. The dilution curve plateaued (Supplementary Figure S1), indicating that the sequencing depth was sufficient to capture the majority of microbial diversity, ensuring the reliability of downstream analyses.

**Figure 2:**
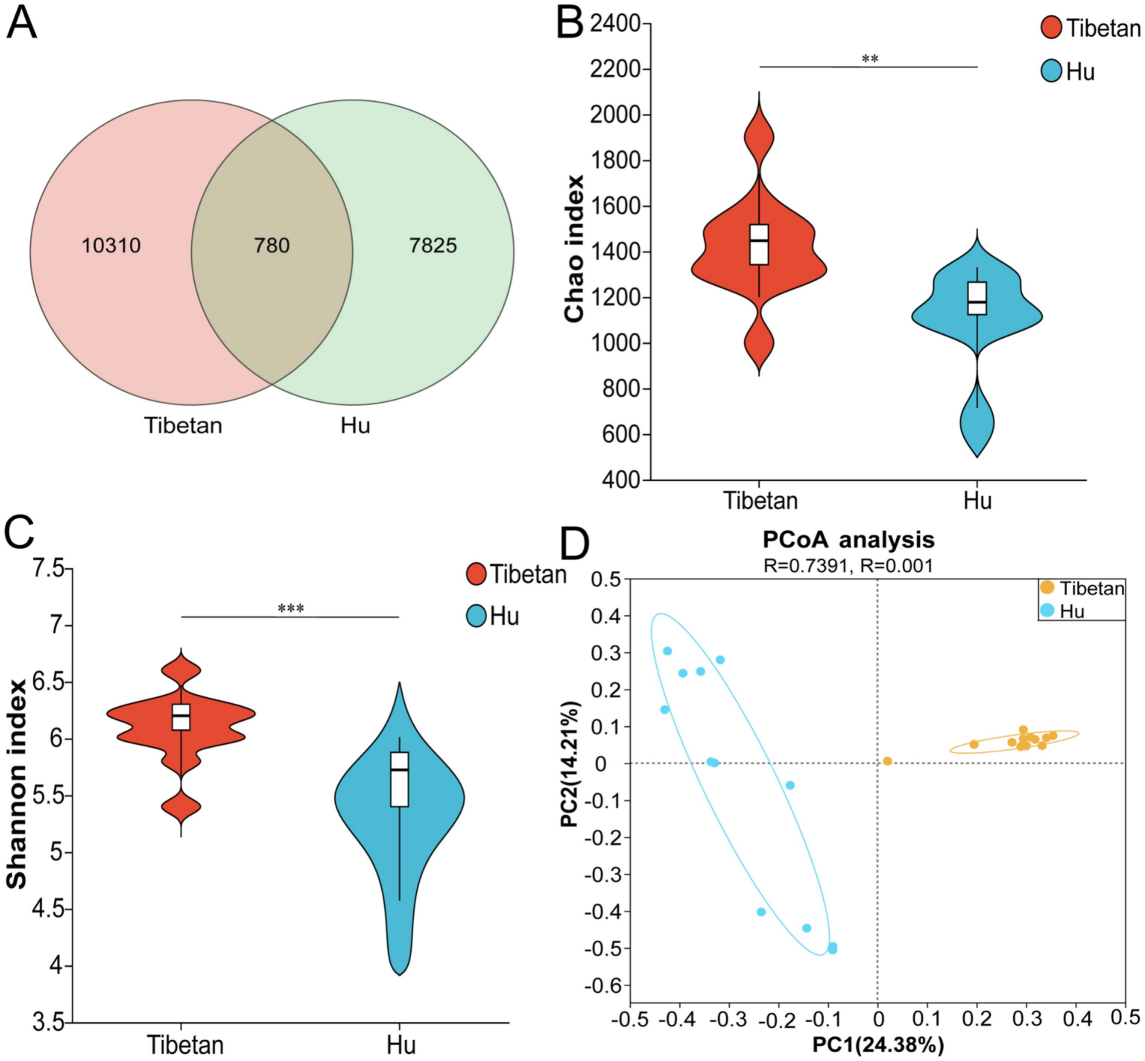
Community diversity characterization. **(A)** Venn diagram showing amplicon sequence variants (ASVs). **(B)** Violin plots of the Chao1 index. **(C)** Violin plots of the Shannon index. **(D)** Principal coordinates analysis (PCoA) at the genus level. **p < 0.01, and ***p < 0.001.

In total, 118,915 amplicon sequence variants (ASVs) were identified across all samples, including 10,310 unique to Tibetan sheep, 7,825 unique to Hu sheep, and 780 shared between both groups (Figure 2A). Alpha diversity analysis revealed that the Chao1 index was significantly higher in Tibetan sheep than in Hu sheep (*p* < 0.05), indicating greater species richness (Figure 2B; Supplementary Table S2). Similarly, the Shannon index was significantly higher in Tibetan sheep, reflecting greater microbial diversity and evenness (Figure 2C). Principal coordinate analysis (PCoA) based on Bray–Curtis distances showed a clear separation between the two groups along PC1 (24.38%) and PC2 (14.21%), indicating significant differences in microbial community structure between Tibetan and Hu sheep (Figure 2D).

### 3.4 Taxonomic composition and differential species of cecal microbiota

**Figure 3:**
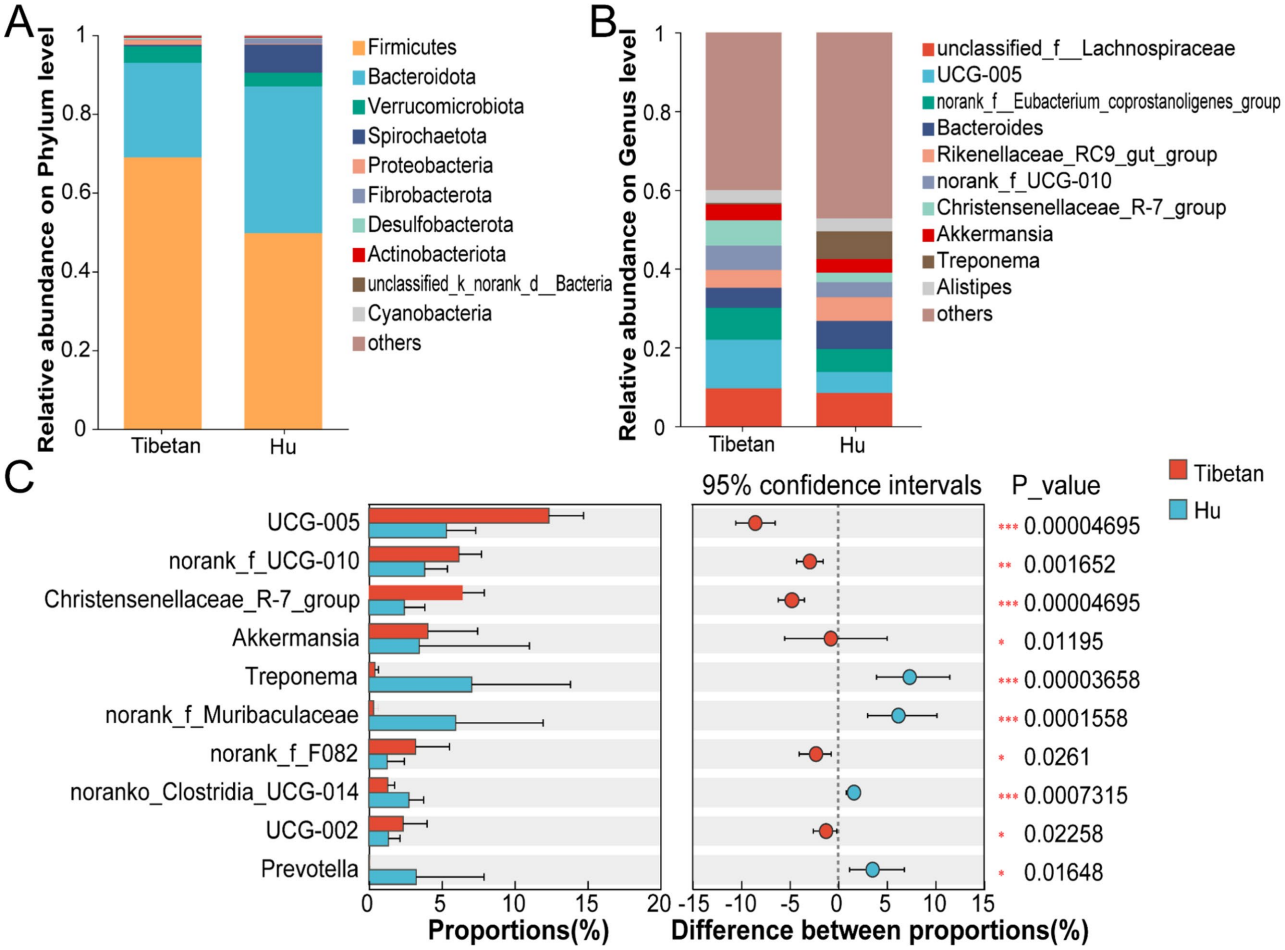
Taxonomic composition and differential analysis. **(A)** Stacked bar plots of taxonomic composition at the phylum level. **(B)** Stacked bar plots of taxonomic composition at the genus level. **(C)** Wilcoxon rank-sum test at the genus level. *p < 0.05, **p < 0.01, and *p* < 0.001.

In order to conduct a more in-depth investigation into compositional disparities in the cecal microbiota between Tibetan sheep and Hu sheep, analyses were conducted at the phylum and genus levels. The results indicate that, at the phylum level, the dominant bacterial phyla in the cecum were Firmicutes (Tibetan: 68.98%, Hu: 49.78%) and Bacteroidetes (Tibetan: 24.03%, Hu: 37.21%) (Figure 3A). At the genus level, *norank_f__Lachnospiraceae*, *UCG-005*, *norank_f__Eubacterium_coprostanoligenes_group*, and *Bacteroides* were the dominant genera shared by both groups. *Rikenellaceae_RC9_gut_group* and *Bacteroides* had higher relative abundances in Hu sheep but the difference was not significant (*p* > 0.05). However, the relative abundance of *Christensenellaceae_R-7_group* and *norank_f__UCG-010* was significantly higher in Tibetan sheep than in Hu sheep (*p* < 0.05) (Figure 3B).

The Wilcoxon rank-sum test further revealed significant differences in the relative abundance of specific genera between the two groups. The abundance of bacterial genera such as *UCG-005*, *norank_f__UCG-010*, and *Christensenellaceae_R-7_group* was significantly higher in Tibetan sheep than in Hu sheep (*p* < 0.05). In contrast, the relative abundance of bacterial genera such as *Treponema*, *Prevotella*, and *norank_f__Muribaculaceae* was higher in Hu sheep (*p* < 0.05) (Figure 3C).

### 3.5 LEfSe biomarker discovery and functional prediction of microbiota

**Figure 4:**
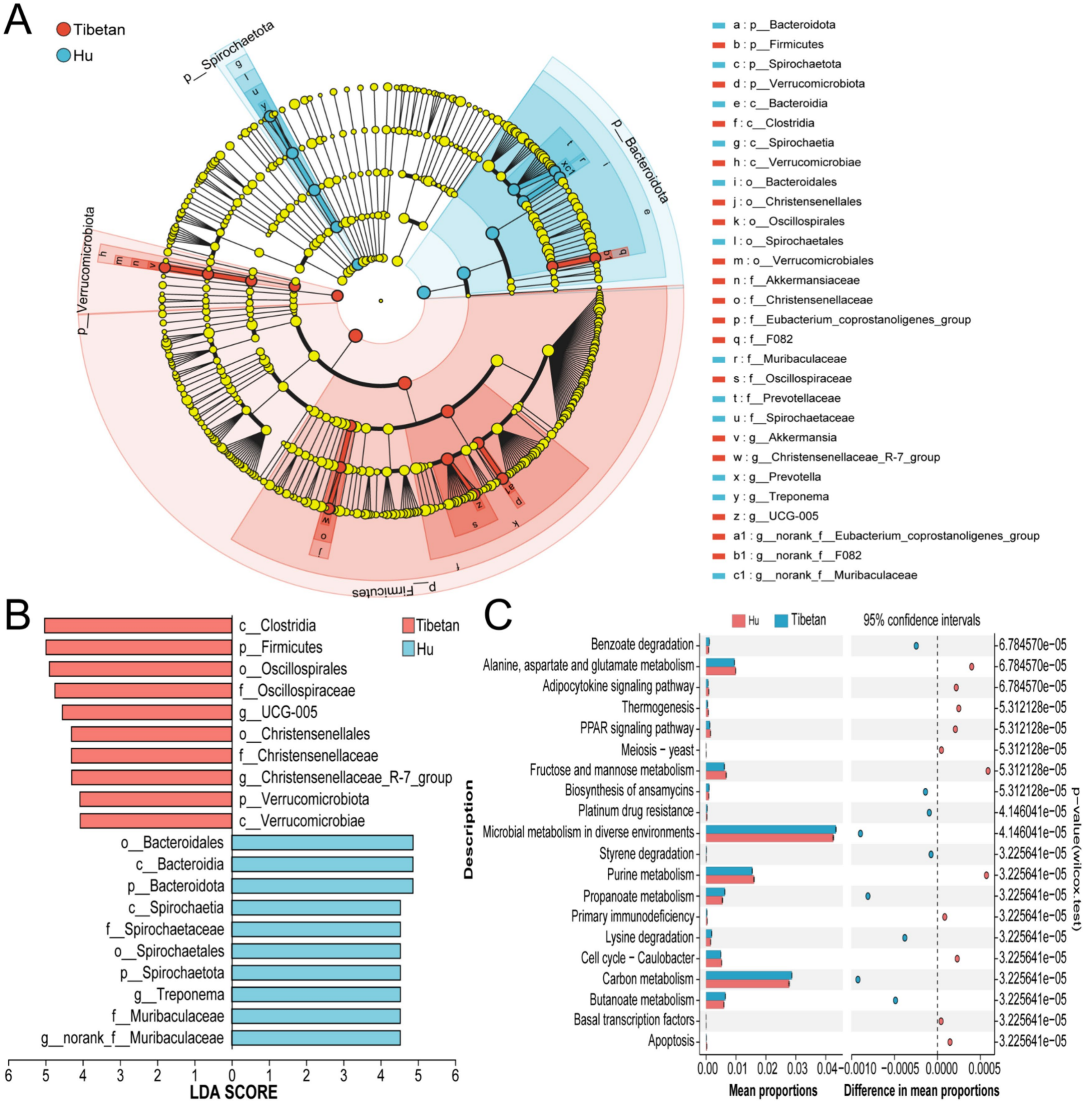
Functional prediction and biomarker identification. **(A)** LEfSe analysis (linear discriminant analysis effect size) from phylum to genus levels. **(B)** Linear discriminant analysis (LDA) score plot showing differential biomarkers. **(C)** STAMP analysis based on PICRUSt2 functional predictions.

LEfSe analyses revealed significant differences in microbial composition between the two groups. At the phylum level, six bacterial phyla with relative abundances greater than 0.5% and LDA scores above 2.0 in at least two samples showed significant differences between the Tibetan and Hu sheep (Supplementary Figure S2). Specifically, Bacteroidota and Spirochaetota were more abundant in Hu sheep, whereas Firmicutes and Verrucomicrobiota were predominant in Tibetan sheep. At the genus level, 53 genera showed relative abundances >1.0% in at least one sample and LDA scores >2.0. Among these, 28 genera were enriched in Hu sheep and 25 in Tibetan sheep (Figure 4A). Further filtering based on LDA scores >4.0 revealed the most significantly differentiated genera. The results showed that *Treponema* and *norank_f_Muribaculaceae* had higher relative abundances in Hu sheep than in Tibetan sheep, while *Christensenellaceae_R-7_group* and *UCG-005* had higher relative abundances in Tibetan sheep (Figure 4B).

The results of the functional prediction, based on the KEGG pathways, indicate that both sheep cecal microbiomes are significantly enriched in multiple key metabolic pathways, including alanine, aspartate, and glutamate metabolism, lysine degradation, propionate metabolism, and butyrate metabolism (Figure 4C). These pathways have been demonstrated to be closely associated with energy metabolism and VFA production, thus suggesting the possibility of differences in microbial metabolic functions between the two sheep breeds.

### 3.6 Non-targeted metabolomics profiling and pathway enrichment analysis

**Figure 5:**
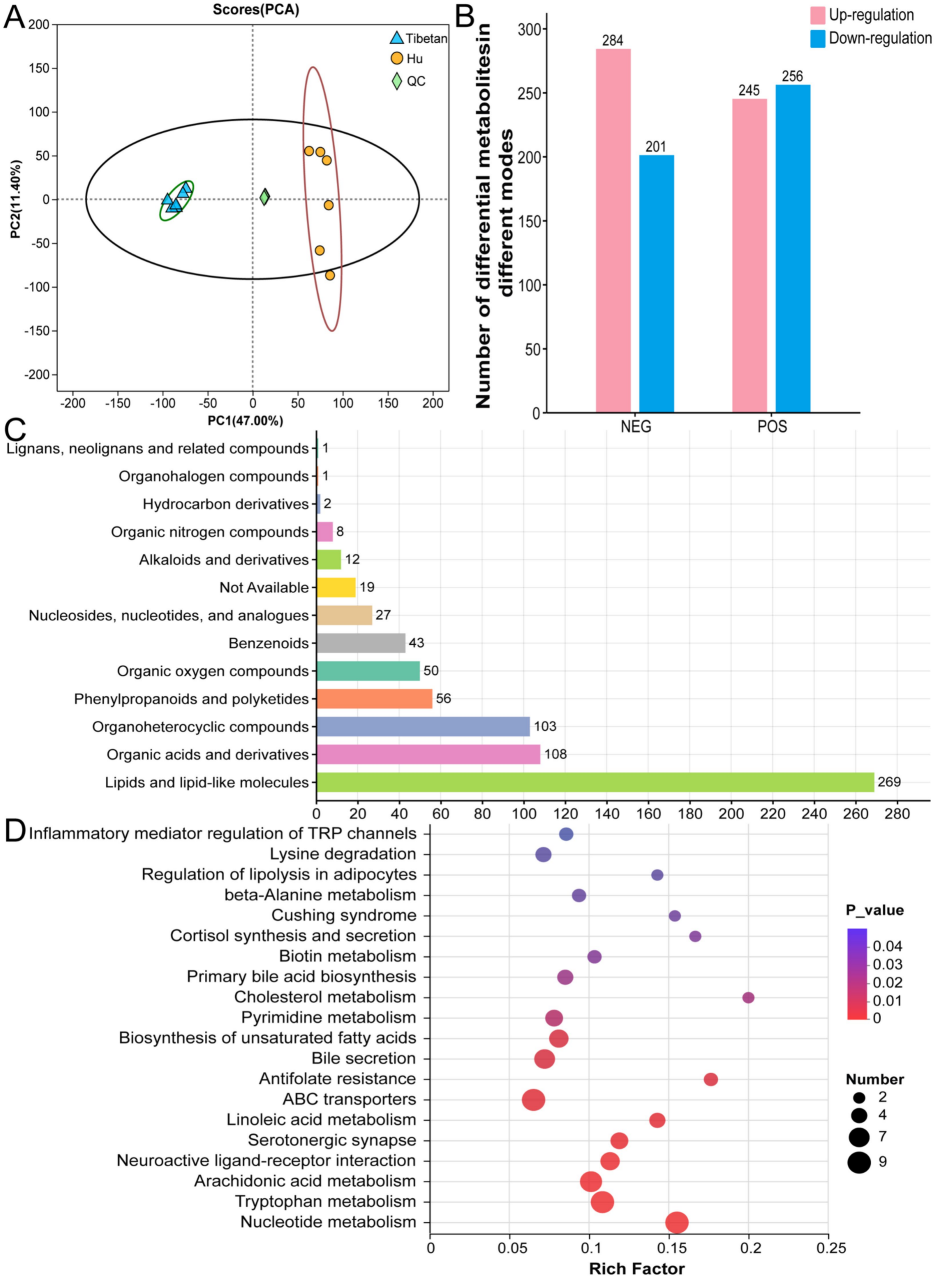
Untargeted metabolomics analysis. **(A)** Principal component analysis (PCA). **(B)** Number of differential metabolites detected across ion modes, including upregulated and downregulated metabolites. **(C)** Classification of differential metabolites. **(D)** KEGG pathway enrichment analysis of differential metabolites.

To investigate differences in intestinal metabolism between Tibetan and Hu sheep, non-targeted metabolomic profiling of cecal contents was performed using liquid chromatography–mass spectrometry (LC–MS). Principal component analysis (PCA) revealed a clear separation between the metabolite profiles of Tibetan and Hu sheep, indicating distinct metabolic compositions between the two groups (Figure 5A).

A total of 986 differentially expressed metabolites (*p* < 0.05, VIP > 1) were detected, including 501 in the positive mode (245 upregulated, 256 downregulated) and 485 in the negative mode (284 upregulated, 201 downregulated) (Figure 5B). Of the differentially expressed metabolites, 699 were successfully annotated using the HMDB. Of these, lipids and lipid-like molecules represented the largest category (269), followed by organic acids and derivatives (108), and organic heterocyclic compounds (103) (Figure 5C; Supplementary Table S3).

KEGG functional annotation classified 171 differentially expressed metabolites into 28 metabolic pathways, involving key biological processes such as lipid metabolism, amino acid metabolism, and coenzyme metabolism (Supplementary Figure S3). Further KEGG pathway enrichment analysis identified 97 enriched pathways associated with differential metabolites, among which 20 were significantly enriched, including core metabolic pathways such as nucleotide metabolism, tryptophan metabolism, and arachidonic acid metabolism (Figure 5D). These findings suggest the possibility of disparities in energy and signaling molecule metabolism between Tibetan and Hu sheep.

### 3.7 Integrative correlation analysis between microbiota and differential metabolites

To explore the potential association between differential metabolites and the microbial community in the cecum, we first excluded unknown differential metabolites and microorganisms with relative abundances below 0.1 to improve the stability and biological interpretability of the analysis. Subsequently, an orthogonal two-dimensional partial least squares (O2PLS) model was used to analyze the interactive effects between the microbiome and metabolome, identifying key metabolites and microbial genera with synergistic trends.

**Figure 6:**
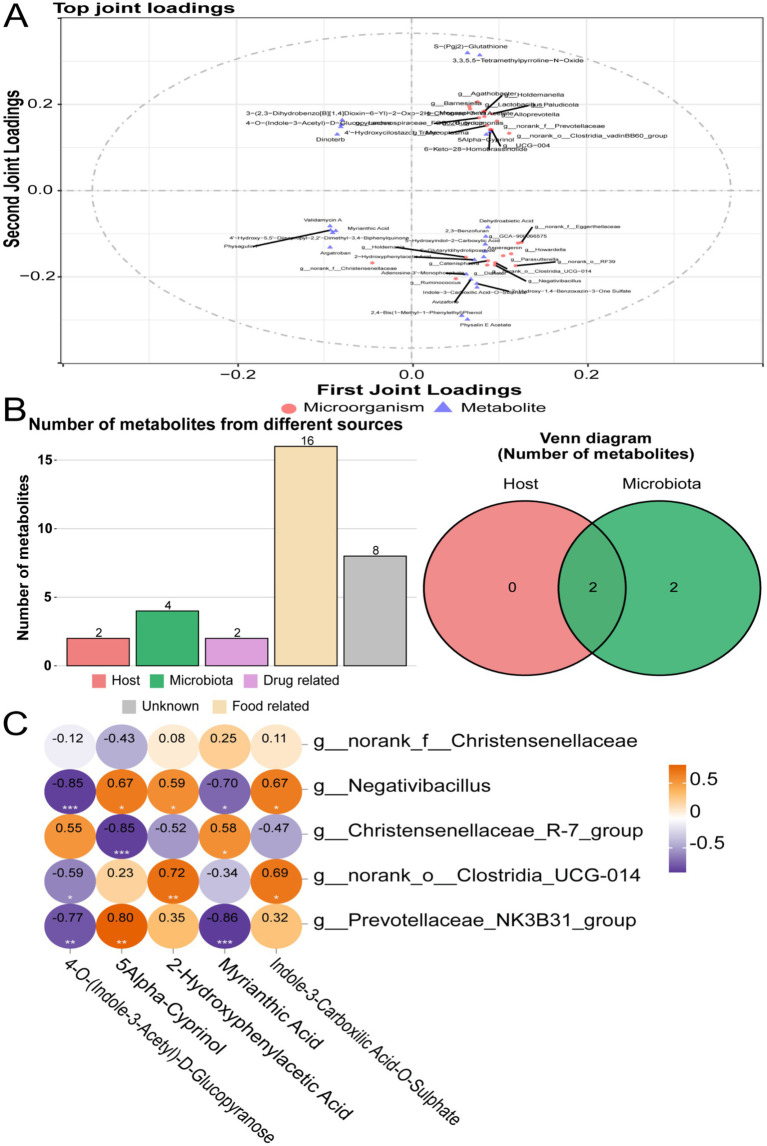
Integrated microbiome-metabolome analysis. **(A)** O2PLS (orthogonal two-way partial least squares) analysis of microbiome and metabolome data. **(B)** Metabolite pathway enrichment analysis (MPEA) of the top 25 metabolites: left, metabolite source analysis; right, Venn diagram showing host and microbial sources. **(C)** Correlation between target metabolites and microbial taxa. *p < 0.05, **p < 0.01, and ***p < 0.001.

O2PLS results revealed significant associations between the top 25 differentially expressed metabolites and microbial genera (Figure 6A). In the upper right quadrant (PC1+/PC2+), *Agathobacter* and *Lactobacillus* co-clustered with metabolites including S-(Pgj2)-Glutathione and 3,3,5,5-Tetramethylpyrroline-N-oxide. In contrast, the lower left quadrant (PC1−/PC2−) featured *Ruminococcus* and *Negativibacillus* co-located with Indole-3-carboxylic acid-O-sulfate and Physalin E acetate.

To further trace metabolite origins, the MetOrigin platform was used for metabolite origin analysis (MPEA), classifying them as host-derived, microbial-derived, food-derived, or of mixed origin. Among the top 25 key metabolites, four were identified as microbial-derived, two as host-derived, and two as co-produced by both host and microbes (Figure 6B).

From the top 25 metabolites, five differentially expressed metabolites with |log_2_ FC| > 2 were screened, including 4-O-(Indole-3-acetyl)-D-glucopyranose, 5Alpha-Cyprinol, 2-hydroxyphenylacetate, myrianthic acid, and indole-3-carboxylic acid-O-sulfate. Among them, 4-O-(Indole-3-acetyl)-D-glucopyranose was significantly upregulated in Tibetan sheep. Among the top 25 differentially expressed microorganisms, five key bacterial genera with *p* < 0.05 were selected for further correlation analysis, including *norank_f__Christensenellaceae*, *Negativibacillus*, *Christensenellaceae_R-7_group*, *norank_o__Clostridia_UCG-014*, and *Prevotellaceae_NK3B31_group*. Among these, *norank_f__Christensenellaceae* and *Christensenellaceae_R-7_group* were significantly more abundant in Tibetan sheep (*p* < 0.05). Finally, correlation analysis confirmed the associations between specific metabolites and microbial genera. 4-O-(Indole-3-acetyl)-D-glucopyranose was significantly positively correlated with *Christensenellaceae_R-7_group* (Figure 6C). These findings suggest the existence of functional synergistic interactions between specific microbial communities and metabolites.

### 3.8 Correlation analysis of microorganisms, metabolites, and growth performance

**Figure 7:**
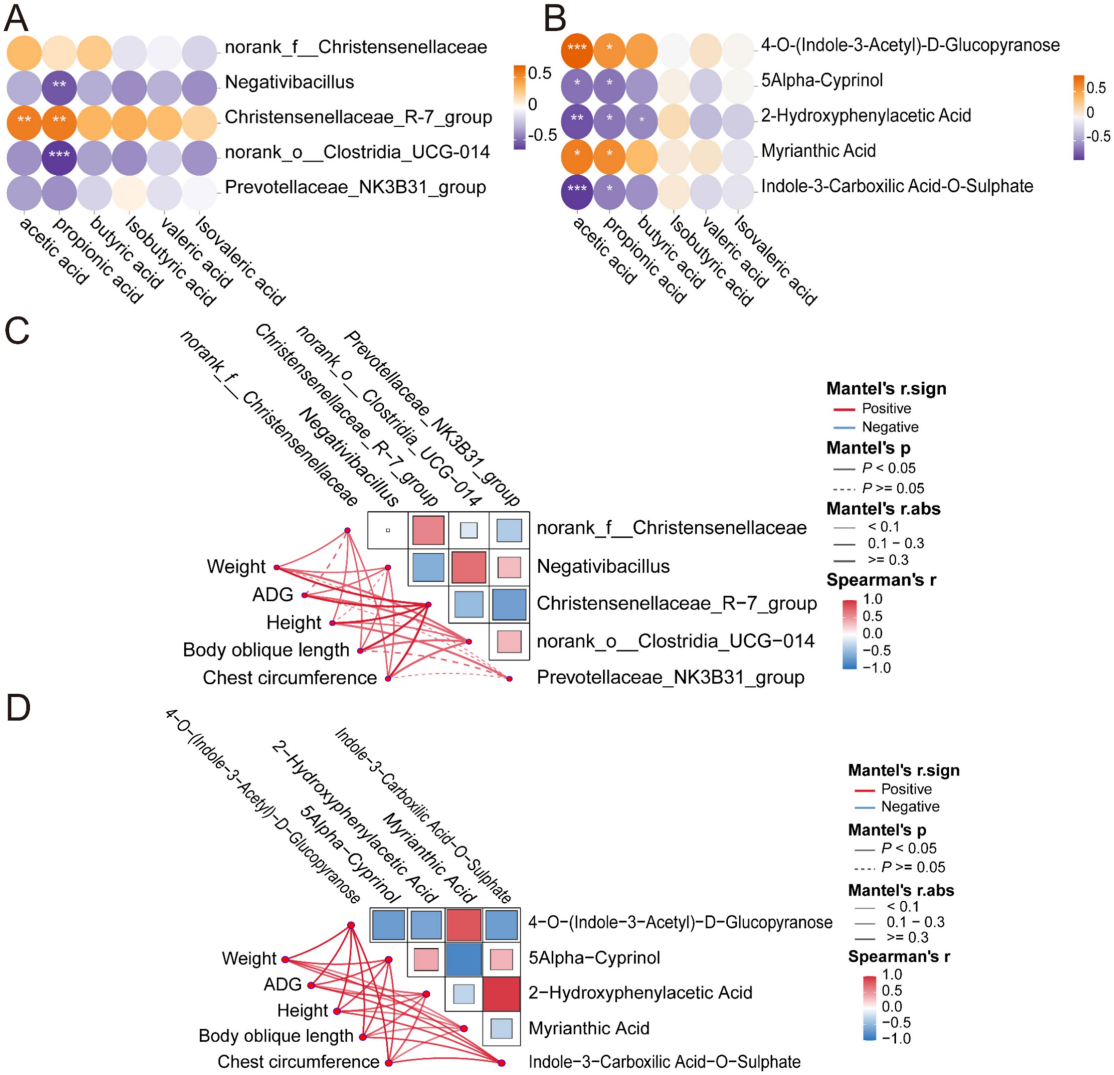
Correlation analysis between microorganisms, metabolites, and production performance. **(A)** Correlation between microbial taxa and VFAs. **(B)** Correlation between metabolites and VFAs. **(C)** Correlation analysis between microorganisms and production performance. **(D)** Correlation analysis between metabolites and production performance. *p < 0.05, **p < 0.01, and ***p < 0.001.

Among the five dominant bacterial groups, *Christensenellaceae_R-7_group* was positively correlated with acetate and propionate, whereas *norank_o__Clostridia_UCG-014* and *Negativibacillus* were negatively correlated with propionate (Figure 7A).

Correlation analysis between differential metabolites and VFAs revealed distinct association patterns with the major VFAs—acetate, propionate, and butyrate. Specifically, 4-O-(indole-3-acetyl)-D-glucopyranose and myrianthic acid showed strong positive correlations with acetate and butyrate, whereas 5Alpha-Cyprinol, 2-hydroxyphenylacetic acid, and indole-3-carboxylic acid-O-sulfate were negatively correlated with both; propionic acid also showed a significant negative correlation with 2-hydroxyphenylacetic acid (Figure 7B).

In order to further investigate the effects of key metabolites and microorganisms on sheep growth performance, correlation analyses were conducted between metabolites, microorganisms, and production traits. The results demonstrated that the five microbial groups were positively correlated with body weight, ADG, body height, body length, and chest circumference (*r* > 0.3; Figure 7C). Notably, *Christensenellaceae_R-7_group* showed a strong positive correlation with ADG (*r* > 0.5), and *Prevotellaceae_NK3B31_group* was positively correlated with body weight and body height (*r* > 0.4). Additionally, five metabolites were also positively correlated with body weight, ADG, body height, body length, and chest circumference (*r* > 0.3; Figure 7D). Among these, 4-O-(Indole-3-Acetyl)-D-Glucopyranose demonstrated the strongest correlations with average daily gain, body length, and girth (*r* > 0.7). In the present study, 5Alpha-Cyprinol demonstrated a robust positive correlation with average daily gain (*r* > 0.6). In addition, Indole-3-Carboxylic Acid-O-Sulfate exhibited significant positive correlations with body weight, body length, and girth (*r* > 0.5). These results suggest that certain microorganisms and their associated metabolites may collectively promote growth performance in sheep.

## 4 Discussion

The digestive tract is the principal site of nutrient absorption, and its digestive enzymes and resident microbiota are key regulators of growth performance. Extensive evidence shows that the gut microbiota significantly influence physiological processes, including nutrient digestion and absorption and overall physiological homeostasis (Zhang et al., 2020; Peixoto et al., 2021; Sha et al., 2024). Moreover, the microbiota contribute to host growth and development, immune regulation, and metabolic function (Dou et al., 2023). Together, these findings provide a mechanistic basis for understanding nutrient utilization in animals and for optimizing husbandry strategies.

This study compared growth performance and cecal fermentation parameters between Tibetan and Hu sheep and revealed significant differences in cecal VFA concentrations between the two breeds. Except for pipe circumference, Hu sheep exhibited significantly greater body weight, ADG, height, body length, and chest circumference than Tibetan sheep. Wang et al. (2023) demonstrated that rumen microbial composition is a key determinant of body weight in Hu sheep. This finding supports our observation that differences in microbial composition among sheep breeds may be associated with superior growth performance by influencing volatile fatty acid production. Similarly, Meng et al. (2024) found that variation in body weight is closely associated with changes in VFA levels along the digestive tract. In ruminants, VFAs are the principal end products of microbial carbohydrate fermentation and represent major substrates for energy metabolism (Vargas et al., 2020). By stabilizing microbial community structure and enhancing fermentation efficiency, VFAs enhance feed digestibility and utilization. These effects improve production traits, including milk yield and meat quality (Huang et al., 2025; Martin et al., 2016). Consistent with these reports, acetic and propionic acid concentrations were significantly higher in the cecum of Hu sheep than in Tibetan sheep in the present study. Velázquez-Cruz et al. (2024) further demonstrated that acetic and propionic acids exert pronounced effects on sheep production performance. Taken together, our findings align well with existing literature and support the view that gut microbiota and volatile fatty acids exert significant effects on sheep production performance.

To identify microbial determinants of VFA differences, this study analyzed the cecal microbiota in two breeds of sheep. In both breeds, Firmicutes and Bacteroidetes dominated the cecal microbiota. However, Tibetan sheep had a higher cecal relative abundance of Firmicutes and a significantly lower abundance of Bacteroidetes than Hu sheep. Firmicutes include taxa that efficiently degrade cellulose and hemicellulose (Zhuo et al., 2020), and enrichment of this phylum is associated with greater fiber-degradation potential and energy yield (Li Q. S. et al., 2022). In contrast, Bacteroidetes preferentially ferment soluble carbohydrates (Ziemer, 2014). Consequently, shifts in these phyla may produce distinct VFA profiles and, in turn, influence production performance. We identified five candidate taxa potentially associated with growth. Among them, *Christensenellaceae*-related taxa (including *Christensenellaceae_R-7_group* and *norank_f__Christensenellaceae*) showed the most consistent associations between growth traits and metabolic indicators. Previous studies report that *Christensenellaceae_R-7_group* is associated with host lipid metabolism and stress resistance (Lu G. et al., 2022). It has also been linked to growth-related phenotypes. Other studies suggest that this taxon influences metabolite-mediated signaling pathways involved in amino acid synthesis and energy metabolism (Ma et al., 2023). These effects may promote amino acid anabolism and lipid metabolism, thereby supporting energy storage and fat deposition. In ruminants, *Christensenellaceae_R-7_group* has been implicated in rumen development and nutrient digestion and absorption (Wei et al., 2024). Higher abundance of this taxon is often associated with increased acetate and butyrate levels (Gebeyew et al., 2021). Similarly, *norank_f__Christensenellaceae* has been linked to fiber degradation and acetate/butyrate production (Leibovitzh et al., 2022). In our dataset, this taxon was positively correlated with acetate and propionate, as well as growth traits (average daily gain, body length, and chest circumference). It was also positively associated with total volatile fatty acids. This taxon was also associated with body height, further supporting its potential relevance to host growth. Collectively, *Christensenellaceae* members (including *Christensenellaceae_R-7_group* and *norank_f__Christensenellaceae*) are consistently linked to cellulose utilization and acetate-related fermentation. In rumen systems, *Christensenellaceae_R-7_group* is positively correlated with acetate concentration and neutral detergent fiber digestibility (Li S. et al., 2022). This pattern suggests a potential role in degrading complex plant carbohydrates, improving substrate conversion efficiency, and generating absorbable end-products such as acetate and butyrate (short-chain fatty acids) that may enhance energy utilization and support host growth. Together, these results support *Christensenellaceae*-related taxa as key links between fiber degradation, SCFA production, and host energy utilization.

*Negativibacillus* was enriched in the sheep cecum and was positively associated with body weight, average daily gain (ADG), body height, body length, and chest circumference. This taxon is reportedly enriched in high-feed-efficiency sheep and has been proposed to contribute to host nutrient metabolism and energy utilization (Zhang et al., 2021). Functionally, *Negativibacillus* has been linked to complex carbohydrate fermentation and may participate in cooperative ecological networks that enhance substrate utilization and hindgut fermentation efficiency (Velasco-Galilea et al., 2021). Collectively, these findings suggest that *Negativibacillus* may support growth by improving nutrient extraction and energy availability, potentially enhancing metabolic efficiency through host–microbe interactions. *Prevotellaceae_NK3B31_group* has been linked to butyrate metabolism and may engage in interspecific competition (Li et al., 2024). In sheep, Cheng et al. (2024) reported that fermented rice husk powder increased the abundance of this group, alongside higher average daily gain and final body weight. In our data, this group was positively associated with growth traits but weakly negatively associated with VFAs, potentially reflecting complex substrate-use strategies and microbial competition. These findings suggest a potential role in maintaining butyrate homeostasis and supporting host growth. *Clostridia* taxa, including *norank_o__Clostridia_UCG-014*, comprise a broad guild of anaerobic Firmicutes involved in glycan utilization and diverse fermentation pathways. *Clostridial* members are widely recognized for harvesting energy from complex dietary glycans and for supporting colonic energy metabolism and intestinal homeostasis, often via butyrate-associated or alternative fermentation routes (Leth et al., 2023). Notably, although *Clostridia_UCG-014* was negatively associated with acetate, propionate, and butyrate in this study, it was positively correlated with body weight, average daily gain, body height, and chest circumference. This pattern suggests that *Clostridia_UCG-014* may redirect substrates toward amino acid metabolism or other energy-yielding pathways rather than directly increasing VFA accumulation (Leth et al., 2023). This metabolic shift may increase the availability of growth-supportive metabolites to the host. Previous studies reported that *Clostridia_UCG-014* responds strongly to dietary interventions and environmental stress, consistent with its potential flexibility in substrate utilization (Wei X. et al., 2025). In addition, clostridial taxa have been linked to production-efficiency metrics in dairy cattle (Marinho et al., 2024). Collectively, these results indicate that *Clostridia_UCG-014* may regulate host–microbiota metabolic interactions and support growth performance through flexible substrate utilization, rather than solely through increased VFA production. Overall, these taxa represent functionally distinct yet complementary guilds that may shape cecal fermentation patterns and energy availability, potentially contributing to breed-specific differences in growth performance.

Metabolomic analysis further revealed key metabolites associated with growth performance, primarily involving tryptophan metabolites and bile acids/sterols. 4-O-(Indole-3-Acetyl)-D-Glucopyranose is a glycosylated derivative of indole-3-acetic acid (IAA), a tryptophan metabolite. As a ligand for the aryl hydrocarbon receptor (AhR), IAA activates the AhR signaling pathway, modulates mucosal immunity, promotes epithelial cell renewal, and maintains host metabolic homeostasis (Ding et al., 2024). Additionally, indole-3-carboxylic acid O-sulfate, a tryptophan-derived metabolite, exhibits notable biological activity (Lu X. et al., 2022). It promotes the growth of beneficial taxa and modulates VFA production by altering the composition and metabolic activity of the gut microbiota (Meng et al., 2024). These observations suggest that the tryptophan pathway may play a central role in regulating digestive function and energy allocation in sheep. Supplementation with 5-hydroxytryptophan (5-HTP) has been reported to improve rumen fermentation efficiency and optimize microbial composition, thereby promoting weight gain (Sun et al., 2024). In this study, 4-O-(indole-3-acetyl)-D-glucopyranose and indole-3-carboxylic acid O-sulfate were positively associated with body weight, ADG, body height, body length, and chest circumference, supporting their potential growth-promoting roles. Collectively, these tryptophan-derived indole metabolites may enhance growth performance by improving intestinal immune homeostasis and barrier integrity, thereby reducing the energetic cost of inflammation and allowing greater energy allocation toward growth and tissue accretion.

5Alpha-Cyprinol is a primary bile acid involved in the emulsification, digestion, and absorption of lipids (Kurogi et al., 2017). In this study, it was positively associated with ADG but negatively associated with acetate, propionate, and butyrate. Bile acid metabolism regulates host cholesterol metabolism and lipid absorption and, via ileal absorption and enterohepatic cycling, shapes the composition of the gut microbiota, thereby modulating VFA production (Larabi et al., 2023). Thus, our findings suggest that 5Alpha-Cyprinol may help maintain energy homeostasis through bile acid–microbiota interactions and consequently support growth performance. This inverse association with VFAs may reflect a metabolic trade-off between hindgut fermentation and small-intestinal lipid digestion, indicating that bile acid-related metabolism supports growth through enhanced lipid absorption rather than increased VFA production.

In sheep, 2-hydroxyphenylacetic acid (2-HPAA) was positively associated with growth indicators, including ADG, body height, and chest circumference. As a tyrosine-derived phenylacetate metabolite, 2-HPAA has been linked to several health-promoting effects in animals (Fernández-Jalao et al., 2021). In goats fed high-concentrate diets, phenylacetate concentrations increase and are positively associated with the abundance of specific rumen microbes, including *Oscillospira* and *Akkermansia* (Hua et al., 2017). These taxa may co-metabolize aromatic compounds, shaping the intestinal fermentation milieu and, in turn, modulating VFA profiles (Meng et al., 2024). In our data, myrianthic acid showed strong positive associations with acetate, propionate, and butyrate. As a secondary metabolite, myrianthic acid has been reported to inhibit fatty acid synthase (FAS), thereby influencing lipid metabolism pathways (Capuano et al., 2024). Taken together, these observations suggest that myrianthic acid may help regulate energy metabolism. Taken together, these findings suggest that breed-specific growth differences may be shaped by coordinated microbial metabolic outputs rather than isolated metabolites, through combined effects on energy harvest and immune-related energy expenditure.

The cecal microbiota and their metabolites may influence host growth through synergistic interactions. Microbial communities ferment dietary substrates to generate metabolites, including short-chain fatty acids and microbiota-modified bile acids. These metabolites can in turn reshape microbial composition and function, forming a bidirectional regulatory network. This microbiota–metabolite synergy may support growth by maintaining barrier integrity and immune homeostasis while enhancing overall energy efficiency. In summary, modulating microbiota–metabolite interactions may represent a microbiome-based nutritional strategy to enhance growth efficiency in sheep; however, causal relationships require further investigation.

## 5 Conclusion

This study characterized breed-specific differences in cecal microbiota composition, predicted functional potential, and metabolite profiles between Tibetan and Hu sheep, and found that these features were closely associated with growth performance. Hu sheep exhibited superior growth traits and higher cecal VFA concentrations, suggesting enhanced hindgut fermentation capacity. Integrated microbiome-metabolome analyses identified five bacterial genera (*norank_f__Christensenellaceae*, *Negativibacillus*, *Christensenellaceae_R-7_group*, *norank_o__Clostridia_UCG-014* and *Prevotellaceae_NK3B31_group*) and five differentially abundant metabolites (4-O-(Indole-3-Acetyl)-D-Glucopyranose, 5Alpha-Cyprinol, 2-Hydroxyphenylacetic Acid, Myrianthic Acid and Indole-3-Carboxilic Acid-O-Sulfate) that were significantly correlated with growth-related traits. Notably, *Christensenellaceae_R-7_group* may contribute to growth by influencing metabolite profiles linked to energy metabolism and intestinal homeostasis. These results suggest that breed-specific differences in growth performance are associated with distinct cecal microbiota composition and metabolite profiles. Modulating gut microbial communities through optimized nutrition and management may improve growth efficiency in sheep; however, further studies are needed to validate causality and practical applicability.

## Data Availability

The datasets presented in this study can be found in online repositories. The names of the repository/repositories and accession number(s) can be found at: https://www.ncbi.nlm.nih.gov/, PRJNA 1280794.

## Footnotes

1
https://www.omicshare.com

2
https://www.omicstudio.cn

3
https://www.chiplot.online

4
http://ieg4.rccc.ou.edu/mena

## Supplementary material

SUPPLEMENTARY FIGURE S1 16S Shannon dilution curve.

SUPPLEMENTARY FIGURE S2 LEfSe analysis LDA greater than 2.

SUPPLEMENTARY FIGURE S3 KEGG pathway level 2 signaling pathway enriched by metabolites.
