# Supplementary figures and images for: Comparative analysis of cecal microbiota and metabolites in relation to growth performance of Tibetan and Hu sheep
Source: Front Microbiol. 2026 Jan 26;16:1725706. doi: 10.3389/fmicb.2025.1725706 (PMC12883762; doi:10.3389/fmicb.2025.1725706)

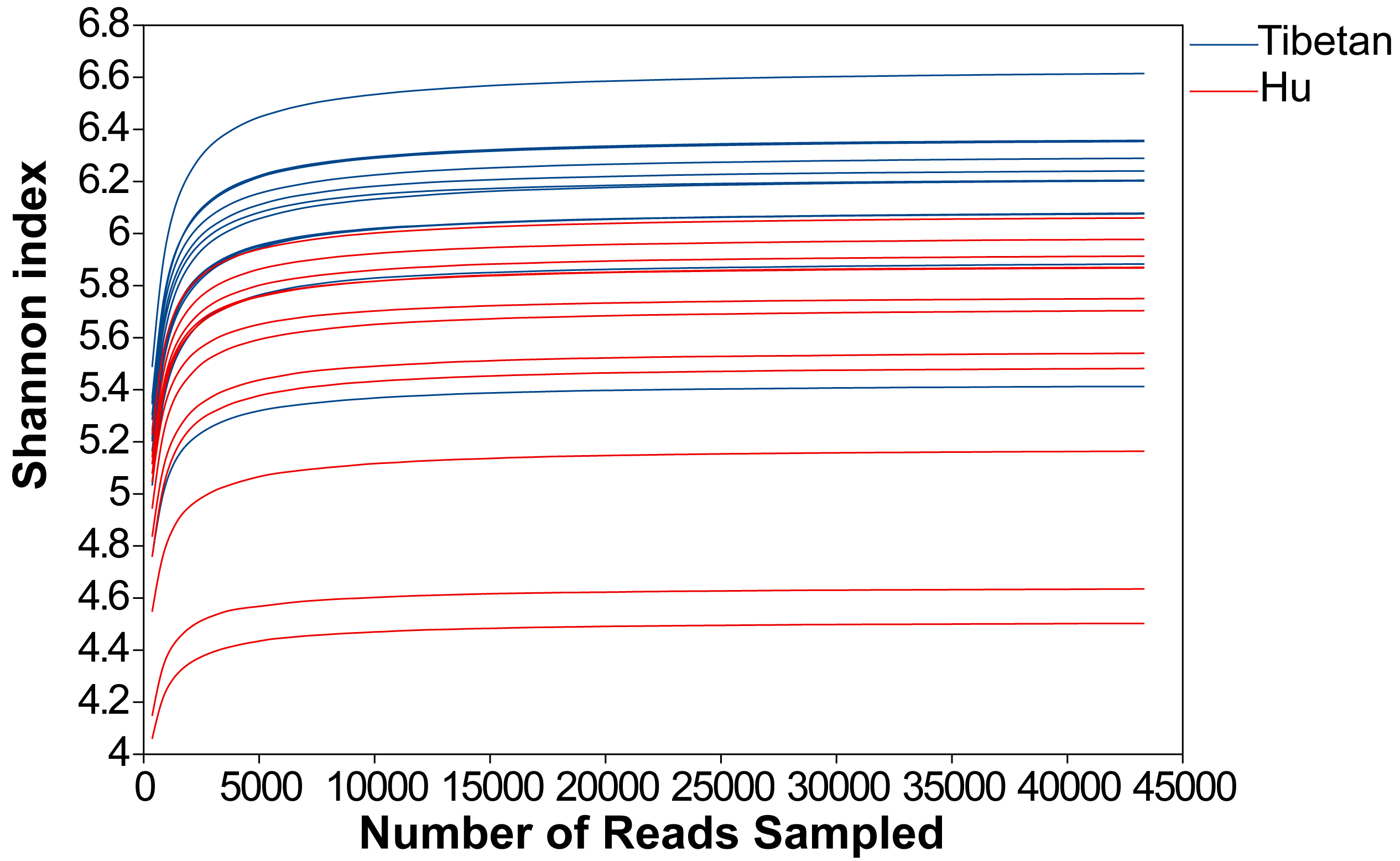

Supplement: SUPPLEMENTARY FIGURE S1 — 16S Shannon dilution curve. [file Image_1.tif]

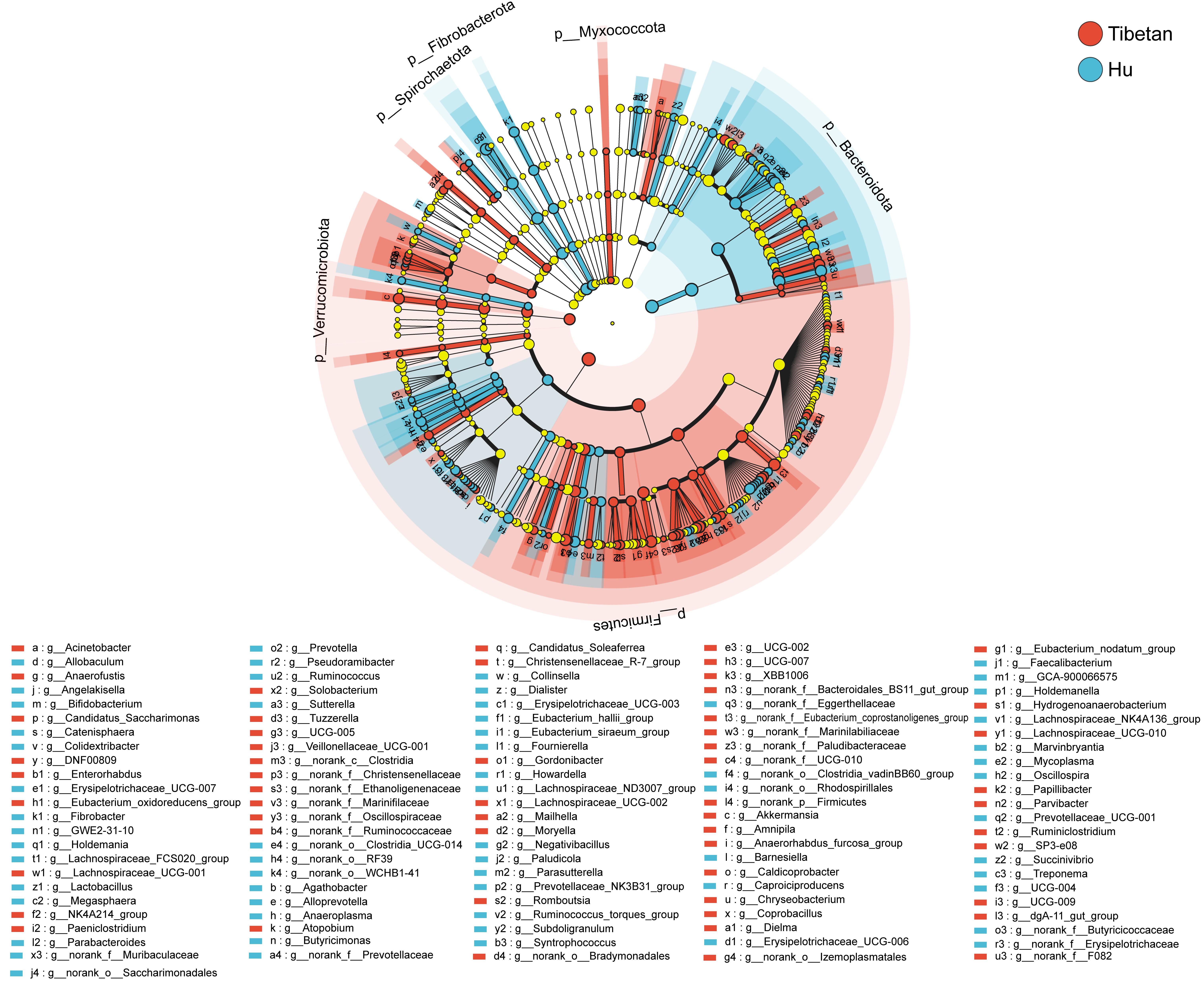

Supplement: SUPPLEMENTARY FIGURE S2 — LEfSe analysis LDA greater than 2. [file Image_2.tif]

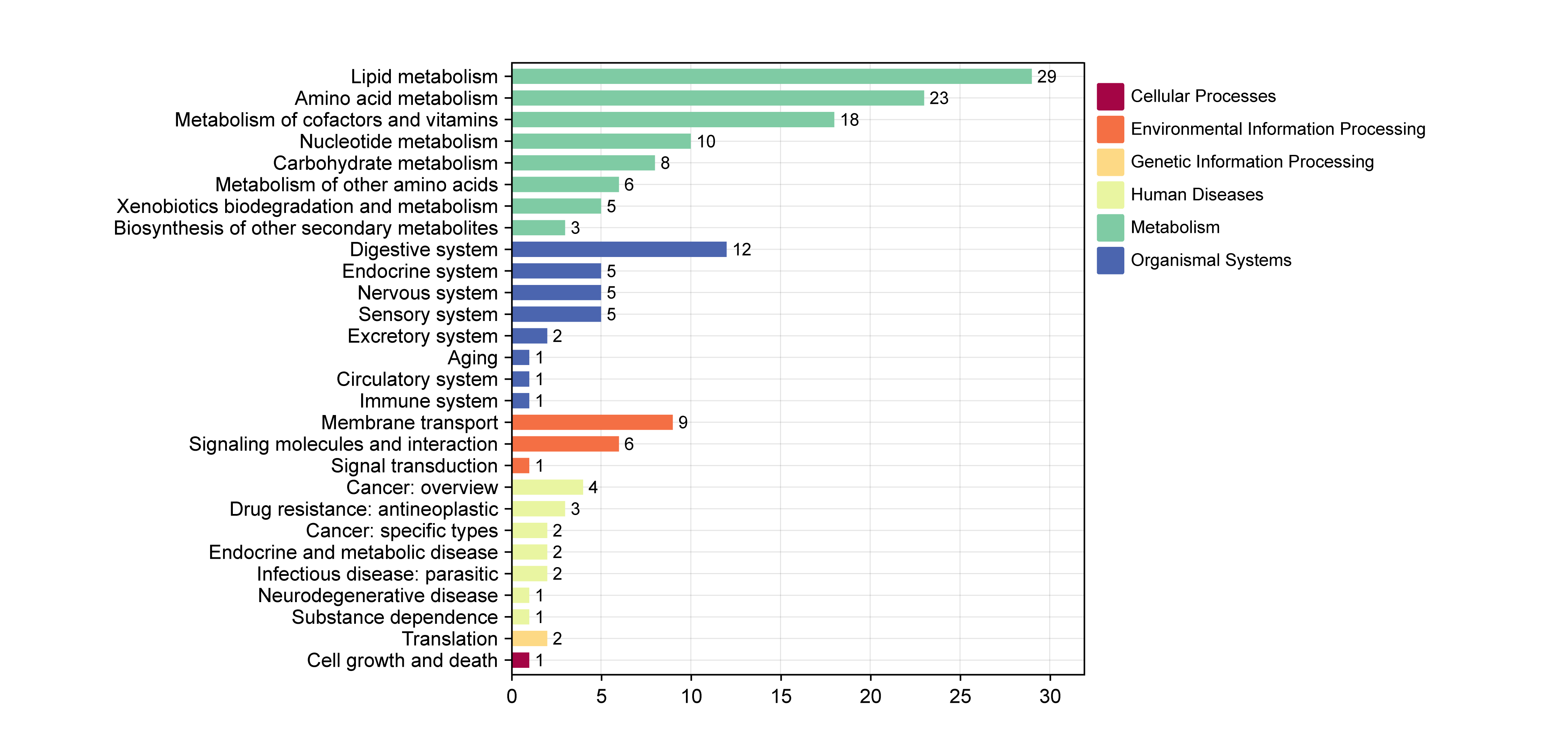

Supplement: SUPPLEMENTARY FIGURE S3 — KEGG pathway level 2 signaling pathway enriched by metabolites. [file Image_3.tif]
